# Genetic engineering of *Trichoderma reesei* cellulases and their production

**DOI:** 10.1111/1751-7915.12726

**Published:** 2017-05-29

**Authors:** Irina S. Druzhinina, Christian P. Kubicek

**Affiliations:** ^1^ Microbiology Group Research Area Biochemical Technology Institute of Chemical, Environmental and Biological Engineering TU Wien Vienna Austria; ^2^ Present address: Steinschötelgasse 7 Wien 1100 Austria

## Abstract

Lignocellulosic biomass, which mainly consists of cellulose, hemicellulose and lignin, is the most abundant renewable source for production of biofuel and biorefinery products. The industrial use of plant biomass involves mechanical milling or chipping, followed by chemical or physicochemical pretreatment steps to make the material more susceptible to enzymatic hydrolysis. Thereby the cost of enzyme production still presents the major bottleneck, mostly because some of the produced enzymes have low catalytic activity under industrial conditions and/or because the rate of hydrolysis of some enzymes in the secreted enzyme mixture is limiting. Almost all of the lignocellulolytic enzyme cocktails needed for the hydrolysis step are produced by fermentation of the ascomycete *Trichoderma reesei* (Hypocreales). For this reason, the structure and mechanism of the enzymes involved, the regulation of their expression and the pathways of their formation and secretion have been investigated in *T. reesei* in considerable details. Several of the findings thereby obtained have been used to improve the formation of the *T. reesei* cellulases and their properties. In this article, we will review the achievements that have already been made and also show promising fields for further progress.

## Introduction

Plant biomass is the most abundant renewable source for conversion to biofuel and biorefinery products. It consists of lignocellulose primary made of various polysaccharides and the aromatic polymer lignin, which provide mechanical strength to the plants and render them highly resistant to attack from pathogens (Houston *et al*., 2016). The main polysaccharides in lignocellulose comprise the glucohomopolysaccharide cellulose (20–50%, w/w) and hemicelluloses (15–35%, w/w), which – depending on the plant–are primarily made up of a xylan, glucuronoxylan, xyloglucan, glucomannan and arabinoxylan backbones with heterogeneous side‐chains (Kubicek, 2013; Álvarez *et al*., 2016).

The use of the monosaccharides that constitute the plant biomass polymers implies their efficient hydrolysis, which – because of its recalcitrance and heterogeneity – is still a major technical challenge. To achieve this, the plant debris have first to be reduced in size by milling or chipping, followed by a mild chemical or physicochemical pretreatment step to make the biomass more susceptible to hydrolysis by the enzymes. Finally, enzymatic hydrolysis depolymerizes cellulose to d‐glucose and the still present hemicelluloses to monosaccharides such as d‐glucose, d‐xylose and l‐arabinose. These two steps are known to be the cost‐intensive part of biomass conversion (Brethauer and Studer, 2015; Payne *et al*., 2015; Gupta *et al*., 2016a; Kubicek and Kubicek, 2016). The current situation and advances in the pretreatment technology have been subject to several excellent recent reviews and shall therefore not be covered here (Xu and Huang, 2014; Silveira *et al*., 2015; Capolupo and Faraco, 2016; Rabemanolontsoa and Saka, 2016). As for the enzymatic hydrolysis step, some of the enzymes in the cellulase cocktail have too low catalytic activity, which impacts the amount needed for the saccharification step. In addition, the composition of the secreted enzyme mixture may not be optimal for certain applications because of the presence of some enzymes in limiting amounts (see below for details). Preparation and use of these enzymes are therefore a major cost factor (Chundawat *et al*., 2011; Klein‐Marcuschamer *et al*., 2012; Gupta *et al*., 2016a; Kubicek and Kubicek, 2016). Enzymatic hydrolysis is nevertheless considered the best available procedure because – in contrast to chemical hydrolysis – it does not produce compounds that inhibit the further conversion of the hydrolysate to biofuels and platform chemicals by fermentation (Payne *et al*., 2015).

To remove the bottleneck at the enzymatic hydrolysis step, a significant amount of work has been performed to understand the performance of the respective enzymes (Beckham *et al*., 2014; Harris *et al*., 2014; Payne *et al*., 2015; Kubicek and Kubicek, 2016). As cellulose represents the most recalcitrant material of the plant biomass, and yields just d‐glucose, the preferred monosaccharide for further use, the majority of work has been performed on cellulolytic enzymes. In the microbial world, there are two fundamentally different strategies for the hydrolysis of cellulose (Payne *et al*., 2015): the ‘bound enzyme paradigm’ (cellulosome) that is present in anaerobic microorganisms (Bae *et al*., 2013), and the ‘free enzyme paradigm’ that is used by aerobic microorganisms (Gupta *et al*., 2016a). The latter is responsible for the vast majority of plant cell wall degradation in nature (Kubicek, 2013). Among the organisms capable of doing this, the ascomycete *Trichoderma reesei* (Hypocreales) was the first microorganism selected for closer investigation because of its potential to produce an efficient cellulose‐hydrolysing enzyme mixture (for review on the history of the detection and use of *T. reesei* see Druzhinina and Kubicek, 2016). Despite the fact that other fungi also produce powerful cellulolytic mixtures, which in some cases even exhibit properties superior to those produced by *T. reesei* (see e.g. Gusakov, 2011; Wang *et al*., 2012 for review), *T. reesei* is still almost exclusively used for cellulase production by industry because technologies for its use and handling are based on a seventy years of experience (Bischof *et al*., 2016).

For a long time, the canonical view of action of fungal cellulases was that endoglucanases (EGs; ‘nonprocessive cellulases’) act by cleaving cellulose chains in amorphous regions within the polymers chain, and cellobiohydrolases (CBHs; processive cellulases) hydrolsze cellulose chains at the end and release the disaccharide cellobiose (‘exo‐endo’ model). This disaccharide is then hydrolysed by β‐glucosidases to glucose. According to their structure, these enzymes are grouped into various glycoside hydrolase (GH) families, which are archived in the CAZyme (carbohydrate active enzymes) database (Lombard *et al*., 2014). Cellulases produced by *T. reesei* belong to five GH families (Table 1): endo‐β‐1,4‐d‐glucanases are found in GH5, GH7, GH12 and GH45, and CBHs in GH6 and GH7. GH7 is the only family that contains both CBHs (CEL7A; previously named CBH1) and endo‐β‐1,4‐d‐glucanases (CEL7B, previously named EGL1).

**Table 1:** *Trichoderma reesei* cellulolytic enzymes

Enzyme type	EC number	GH family	Abbreviated name^a^	Full name	Previous name	Protein ID^b^
Cellobiohydrolases	EC 3.2.1.91	GH6	CEL6A	Cellobiohydrolase II	CBHII	Trire2:72567
	EC 3.2.1.91	GH7	CEL7A	Cellobiohydrolase I	CBHI	Trire2:123989
Endo‐β‐1,4‐d‐glucanases	EC 3.2.1.4	GH5	CEL5A	Endoglucanase II	EGL2	Trire2:120312
	EC 3.2.1.4	GH5	NN	Endoglucanase		Trire2:53731
	EC 3.2.1.4	GH5	NN	Endoglucanase		Trire2:82616
	EC 3.2.1.4	GH7	CEL7B	Endoglucanase I	EGLI	Trire2:122081
	EC 3.2.1.4	GH12	CEL12A	Endoglucanase III	EGL3	Trire2:123232
	EC 3.2.1.4	GH12	NN			Trire2:77284
	EC 3.2.1.4	GH45	CEL45A	Endoglucanase V	EGL5	Trire2:49976
β‐d‐glucosidases	EC 3.2.1.21	GH3	CEL3A	β‐glucosidase I	BGL1	Trire2:76672
	EC 3.2.1.21	GH3	CEL3B	β‐glucosidase		Trire2:121735
	EC 3.2.1.21	GH3	CEL3C	β‐glucosidase		Trire2:82227
	EC 3.2.1.21	GH3	CEL3E	β‐glucosidase		Trire2:76227
	EC 3.2.1.21	GH3	CEL3F	β‐glucosidase		Trire2:104797
	EC 3.2.1.21	GH3	CEL3H	β‐glucosidase		Trire2:108671
	EC 3.2.1.21	GH3	CEL3J	β‐glucosidase		Trire2:66832

a NN, no specific name given yet; the GH3 03B2‐glucosidases CEL3D and CEL3G are not listed because they are intracellular enzymes (Guo *et al*., 2016).

b Refers to the *T. reesei* genome database (http://genome.jgi.doe.gov/Trire2/Trire2.home.html).

GH5 cellulases are most abundant in fungi (Li and Walton, 2017), and also three members of this family are present in *T. reesei*. GH7 enzymes are common, and orthologues of CEL7A are the most prevalent cellulolytic enzymes in the secretomes of biomass‐degrading fungi. Because of its processive mode of catalysis (like also CEL6A, which is however usually present in much smaller concentrations), it depolymerizes cellulose most rapidly and is also responsible for the majority of hydrolytic turnover. The GH6 family is currently the only known family that comprises cellulases that act from the nonreducing end of the cellulose chain. CEL7A and CEL6A therefore act in synergism and are considered as primary components in biomass degradation cocktails.

GH12 enzymes are typically characterized by a low molecular weight (25 kDa), and – in contrast to most other cellulases – lack a cellulose‐binding domain (CBM1) and glycosylation. They can therefore diffuse deeper into cellulosic material, which made them preferred candidates for the laundry industry. GH45 cellulases are also generally small but have a broader substrate specificity compared to GH5 and GH7 endo‐β‐1, 4‐d‐glucanases. Interestingly, GH45 enzymes are structurally and evolutionarily related to plant expansins.

*Trichoderma reesei* β‐glucosidases are found in the GH1 and GH3. Those belonging to GH1 are exclusively intracellular enzymes, whereas seven of the nine GH3 β‐glucosidases are secreted into the medium (Guo *et al*., 2016). CEL3A (previously named BGL1) accounts for most of the β‐glucosidase secreted activity.

**Figure 1:**
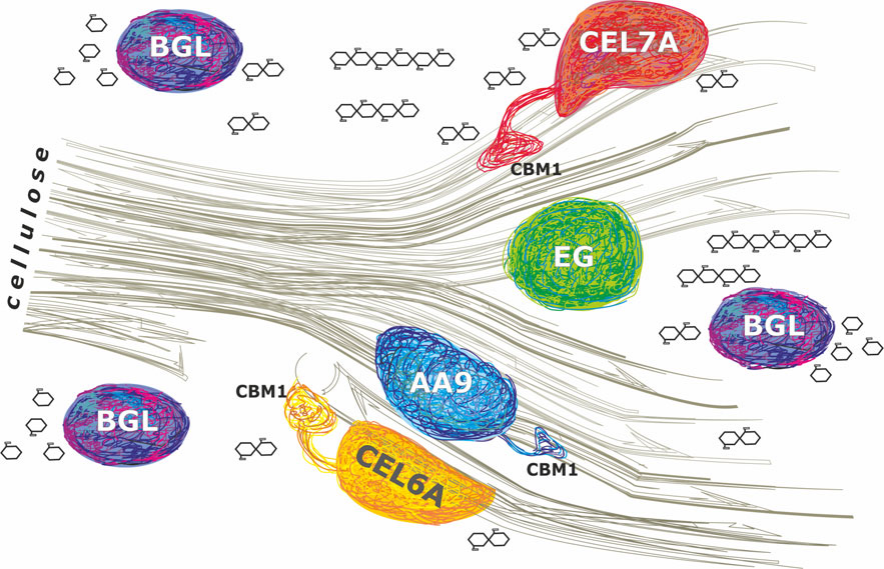
Cartoon summarizing the current knowledge about the *Trichoderma reesei* enzymes that attack and hydrolyse cellulose. Abbreviations: CEL7A cellobiohydrolase CBH1; CEL6A cellobiohydrolase CBH2; CBM1 cellulose‐binding domain, if present; EG, endoglucanase; AA9, lytic polysaccharide monooxygenase; BGL ‐ β‐glucosidase. CBH1 and CBH2 cleave at the (reducing and non‐reducing, respectively) ends of the cellulose chain. EG cleaves in amorphous cellulose regions, AA9 can act on both crystalline and less‐crystalline regions. The oligosaccharides are further hydrolysed to d‐glucose by β‐glucosidase (BGL, EC 3.2.1.21).

The ‘exo/endo’ model (for review see Kubicek, 2013) has been revised by Stahlberg *et al*. (1993) and Kurašin and Väljamäe (2011) who showed that CEL7A is also able to conduct ‘endo‐initiation’ and is therefore not a true exocellulase. However, neither the EGs nor the CBHs from fungi can cause massive cellulose decomposition. They rather ‘peel one layer at a time’ (Payne *et al*., 2015). Elwyn T. Reese and co‐workers had therefore in the fifties already proposed that a decrystallizing protein must exist, which does not hydrolyse the β‐glycosidic linkage but possesses the ability to swell or disrupt cellulose (the so called ‘C1 factor’; Reese *et al*., 1950). The nature of this C1 protein was enigmatic for a long time. The recently detected lytic polysaccharide monooxygenases (LPMO; originally classified as endoglucanases GH61), attack the highly crystalline regions of cellulose and cleave cellulose by oxidation. They could therefore represent the earlier postulated C1 factor (Morgenstern *et al*., 2014; Johansen, 2016a,b). Another *T. reesei* protein, SWO1 which bears an expansin‐like domain has also been considered to fulfill a function in preparing cellulase or hydrolytic attack. But recently published results with SWO1 that had been produced in a cellulase‐negative *T. reesei* strain failed to show a synergy with cellulases in the degradation of pretreated lignocellulose (Eibinger *et al*., 2016). The authors therefore concluded that SWO1 ‘is not a C_1_ factor of degradation of pure cellulose’. A summary of our current understanding of enzymatic cellulose hydrolysis by ‘free cellulases’ is given in Fig. 1).

Not surprisingly, *T. reesei* has been the subject of intensive investigation towards the improvement of its cellulases and reducing the costs for their production and use. This involved attempts to increase their intrinsic activity, facilitating their production, and the reinforcement of existing cellulase preparations by auxiliary proteins and enzymes from other organisms (Wilson, 2009; Horn *et al*., 2012; Peterson and Nevalainen, 2012; Hu *et al*., 2015; Müller *et al*., 2015; Payne *et al*., 2015; Kubicek and Kubicek, 2016). In this review, we will summarize the progress in these fields, thereby focusing exclusively on cellulose hydrolysis by *T. reesei*.

## Protein engineering of *T. reesei* cellulase

Despite the progress that has been made in molecular understanding of the reaction mechanism of several cellulases and their binding to cellulose (for extensive review see Payne *et al*., 2015), these findings have (at least in the published literature) not yet led to strategies for improvement of cellulase activities of *T. reesei* by genetic engineering. Rather, the focus has been put on the stability of the enzymes at increased temperatures, activity in broader pH ranges and the relief from inhibition by components present in the saccharification mixture.

### Engineering high‐temperature tolerance of cellulases

Thermostable cellulases are a major goal in the lignocellulose degradation technology. Performing lignocellulose saccharification at elevated temperatures would provide several benefits such as increased specific activity and stability, prevention of growth of contaminants and increased mass transfer rate due to lower fluid viscosity at high substrate concentrations (Viikari *et al*., 2007; Blumer‐Schuette *et al*., 2014). In addition, the biomass saccharification rate is inhibited by lignin that is still present, but this inhibition is less pronounced in thermostable enzymes (Rahikainen *et al*., 2013). *Trichoderma reesei* is mesophilic and its enzymes are consequently only moderately tolerant to temperatures above 50 °C (Chokhawala *et al*., 2015). While the incorporation of respective cellulase genes from thermotolerant fungi such as *Acremonium thermophilum, Thermoascus aurantiacus, Chaetomium thermophilum, Myceliophthora thermophila*, or *Thielavia terrestris* is an option (Viikari *et al*., 2007; Voutilainen *et al*., 2008), there have also been several attempts to improve the thermostability of *T. reesei* cellulases by protein engineering: Sandgren *et al*. (2003) pioneered this area by comparing the amino acid sequence of CEL12A from *T. reesei* and its thermally much less stable orthologue from *T. citrinoviride*. The two proteins differed only in 14 amino acids, and exchanging each of them in *T. reesei* CEL12A by those from *T. citrinoviride* CEL12A identified an A35S substitution that most strongly decreases the thermal stability. Interestingly, a highly thermostable CEL12A from an unidentified *Streptomyces* sp. displayed an A35V substitution, and the introduction of this A35V mutation into *T. reesei* CEL12A resulted in a T_m_ that was 7.7 °C higher than that of the native enzyme. Mutations of neither A_35_, S_35_ nor V_35_ had an influence on the overall protein structure or hydration of the protein. However, the presence of a hydrophobic amino acid such as A or V at position 35 caused tighter van der Waals interactions with its three neighbouring amino acid side‐chains and closed the entry to the β‐sheet sandwich core. This behaviour, known as cavity‐filling mutation, has been observed in thermal stabilizing mutations in other GHs too (Karshikoff *et al*., 2015).

Lantz *et al*. (2010) demonstrated that the two CBHs CEL6A and CEL7A of *T. reesei* are more thermolabile than the other enzymes involved in cellulose breakdown (like endo‐β‐1,4‐glucanase CEL5A and the β‐glucosidase CEL3A). They are therefore likely rate limiting to the performance of the whole cellulase mixture at saccharification temperatures over 50 °C. Consequently Day *et al*. (2007) compared the amino acid sequence and structure of 42 CEL7A family members from various fungi and identified 19 sites that could be involved in increased thermostability. Using site saturation mutagenesis, they indeed showed that mutations in 18 of these sites enhanced temperature stability, and the introduction of all 18 sites into CEL7A produced a variant, which exhibited an increase in *T*_m_ of 14.8 °C (*T*_m_ 76.0 °C; Lantz *et al*., 2010).

Arnold and colleagues from the California Institute of Technology have made substantial contributions to GH7 engineering primarily on the basis of computational prediction tools such as structure‐guided recombination. They used non‐contiguous recombination (NCR), a method that identifies pieces of structure that can be swapped among homologous proteins to create new chimeric proteins (Smith *et al*., 2013a). By this means they designed a library of chimeric enzymes, in which blocks of the structure from *T. reesei* CEL7A and the two thermostable CEL7A homologues from *Talaromyces emersonii* and *C. thermophilum*, respectively, were shuffled to create 531,438 possible variants (Smith *et al*., 2013b). Selecting a maximally informative subset of 35 chimeras for analysis, they found that these blocks contributed additively to the stability of a chimera. Two highly stabilizing blocks displayed the same two mutations (T360A and F362M), and they increased the thermal stability of CEL6A by 1 and 3°C, respectively (Smith *et al*., 2013b).

### Engineering cellulases for resistance towards ionic liquids

As explained above, the recalcitrant nature of native lignocellulose makes a pretreatment by chemical, physical or biological means necessary. Classical pretreatment procedures, however, are performed in a separate step before hydrolysis, and so necessitate extensive washing (for review see Brethauer and Studer, 2015). Consequently, pretreatment and hydrolysis in a one‐pot reaction would improve the process from an economic point of view. Among the various pretreatment methods, the use of ionic liquids (ILs) would be most appropriate because it does not involve harsh conditions and does not lead to the formation of toxic by‐products (Uju *et al*., 2012). However, the strongly nucleophilic ILs interact with the positively charged residues on the surface of the cellulases and inactivate them (Li *et al*., 2015). A possibility for overcoming this is a chemical modification of the primary amino groups in *T. reesei* CEL7A by succinylation and acetylation, which results in a doubling of the rate of cellulose hydrolysis in 15% (v/v) of the IL 1‐butyl‐3‐methylimidazolium chloride (BMIM‐Cl) (Xu *et al*., 2016). ILs can also strongly bind to the cellulose‐binding tunnel in the catalytic domain of CEL7A (Li *et al*., 2015), and interrupt binding of cellulose to the hydrophobic amino acids that are located on the flat face of CBM1 (Wahlstrom *et al*., 2014). Li *et al*. (2015) identified six amino acids near the active site that strongly bind the 1‐butyl‐3‐methylimidazolium cation (BMIM]^+^) and provided *in silico* evidence that mutation of these residues reduced [BMIM]^+^ binding and enhanced the tolerance to ILs. However, the production of such a mutated enzyme by *T. reesei* has not yet been documented.

### Manipulation of the enzyme composition

The oldest tool to manipulate the enzyme spectrum produced by *T. reesei* is the introduction or deletion of one or more cellulase or hemicellulase genes. Soon after the development of the first transformation systems for *T. reesei* (Penttilä *et al*., 1987; Gruber *et al*., 1990; Smith *et al*., 1991), first successful attempts in this direction had been published (Harkki *et al*., 1991; Kubicek‐Pranz *et al*., 1991). Manipulation or elimination of the production of certain cellulase components is particularly important for the laundry and textile industry, where they can damage tissues (Galante *et al*., 1998).

Another limitation of the *T. reesei* cellulase system is its low activity of β‐glucosidase, which leads to accumulation of cellobiose during biomass hydrolysis. This in turn inhibits cellobiohydrolase and endo‐β‐1,4‐glucanase activities and so reduces the saccharification rate (Gruno *et al*., 2004). This low β‐glucosidase activity is not due to too low expression of the respective genes but because the main extracellular β‐glucosidase – CEL1B – remains trapped to a high percentage within the fungus’ cell wall and only part of it slowly released into the medium during autolysis (Kubicek, 1981). Although the cell wall components responsible for this trapping have been identified (Rath *et al*., 1995), there have been no attempts to use this information for producing strains that secrete more CEL1B into the medium. Instead, this β‐glucosidase deficiency was compensated by the introduction of β‐glucosidase genes from other fungi into *T. reesei*. Various donors have been reported, such as *Penicillium decumbens* (Ma *et al*., 2011), *Aspergillus aculeatus* (Nakazawa *et al*., 2012; Treebupachatsakul *et al*., 2015), a *Periconia* sp. (Dashtban and Qin, 2012), *Rasamsonia emersonii* (Ellilä *et al*., 2017), *Neosartorya fischeri* (Xue *et al*., 2016) and *Chaetomium atrobrunneum* (Colabardini *et al*., 2016). The enzymes from the last three thermotolerant species also are more stable at higher temperatures, thereby removing the limitation of saccharification by the low‐temperature stability of *T. reesei* CEL1B during hydrolysis (Viikari *et al*., 2007). To this end, Xue *et al*. (2016) reported that a fusion of the *N. fischeri* NfBgl3A gene to the *cbh1* structural gene resulted in β‐glucosidase activities that were up to 175‐fold higher than those of the parent strain.

Cellobiose dehydrogenase (CBD, EC 1.1.99.18), a member of the enzyme arsenal auxiliary to glycoside hydrolases (family AA3; Levasseur *et al*., 2013), is an extracellular enzyme produced by various wood‐degrading fungi (Henriksson *et al*., 2000). It oxidizes the reducing ends of cellobiose and cellooligosaccharides to 1, 5‐lactones, which are subsequently hydrolysed to the corresponding carboxylic acids. CBD orthologues’ have also been found in several Pezizomycotina genomes (Table S1). Although *T. reesei* contains several members of the AA3 family too (Druzhinina and Kubicek, 2016) – a CBD orthologue is absent. Ayers *et al*. (1978) first suggested that CBD (named ‘cellobiose oxidase’ then) from *Phanerochaete chrysosporium* could be involved in cellulose biodegradation. Bey *et al*. (2011) demonstrated that the addition of CBD from the basidiomycete *Pycnoporus cinnabarinus* synergistically stimulated the saccharification of wheat straw by a *T. reesei* commercial cellulase cocktail. This stimulation is due to relieving CBH1 from the competitive inhibition by cellobiose, which is oxidized by CBD to the non‐inhibitory cellobionolactone. Consequently, Wang and Lu (2016) overexpressed a CBD‐encoding gene from *P. chrysosporium* under the *cbh2* promoter in *T. reesei*, and obtained transformants with up to fourfold increased filter paper activity.

The identification of LPMOs as a group of enzymes that accelerate the breakdown of carbohydrate polymers like cellulose, chitin and starch by oxidative cleavage has been a breakthrough in lignocellulose conversion research (Johansen, 2016a,b). LPMOs belonging to the auxiliary enzyme family AA9 degrade cellulose nanofibrils on the surface into shorter fragments. They therefore assist cellulases to attack otherwise highly resistant crystalline substrate areas, which results in a faster and more complete surface degradation (Eibinger *et al*., 2014). Supplementation of the commercial cellulase cocktail Cellic CTec1 (Novozymes) with AA9 enzymes improved the hydrolysis of lignocellulose (Sun *et al*., 2015), which led to the new commercial cellulase preparations Cellic CTec2 and Cellic CTec3.

Because of the role of CBD in assisting the catalysis by AA9, it is possible that this stimulatory action of CBD (*vide supra*) is due to the stimulation of AA9. *Trichoderma reesei* has three AA9‐encoding genes, of which one is strongly induced during growth under cellulase inducing conditions. The enzyme has been overproduced in *T. reesei* (Karlsson *et al*., 2001), but the resulting cellulose‐hydrolysing activity of the secreted cellulolytic enzymes has not been identified (at that time, the enzyme was considered to be an endo‐β‐1,4‐glucanase, CEL61A). The successful overexpression of AA9 – together with CBD – in *T. reesei* may directly lead to strains with improved cellulase performance.

Finally, *T. reesei* lacks invertases that hydrolyse sucrose (Bergès *et al*., 1993), and therefore cannot grow on some cheap technical substrates such as sugarcane molasses. Ellilä *et al*. (2017) showed that this deficiency could be overcome by constitutively expressing an invertase gene from *Aspergillus niger* in *T. reesei*.

## Engineering cellulase gene expression

**Figure 2:**
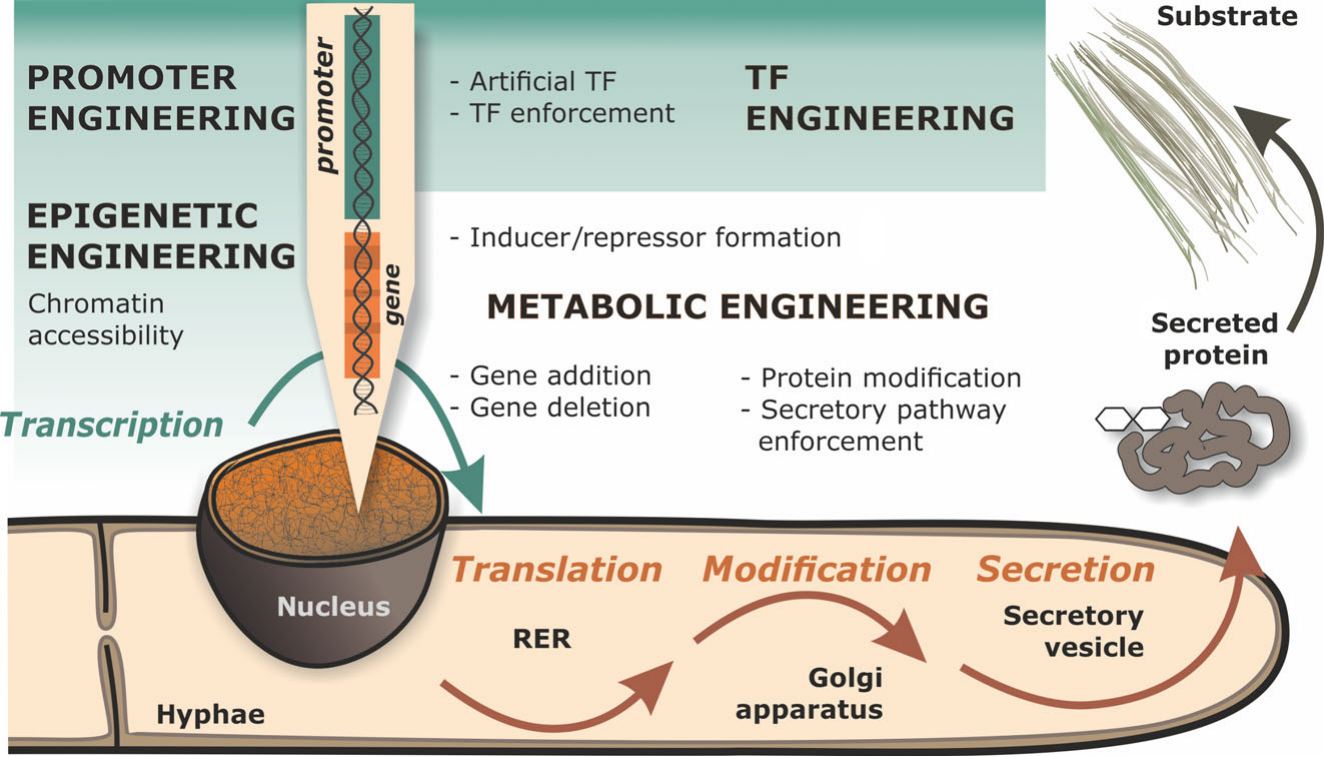
An overview of genetic engineering strategies that offer new perspectives for the further improvement of *Trichoderma reesei* cellulase formation. Abbreviations: TF – transcription factor, RER – rough endoplasmic reticulum.

Various cellular levels have been identified which influence the expression of cellulases (for overview see Fig. 2), which will be described below.

### Genetic engineering of transcriptional regulators

In *T. reesei*, the expression of all cellulases and of the majority of hemicellulases is induced in a coordinated manner by growth on carbon sources such as cellulosic substrates, or some disaccharides such as lactose or sophorose, respectively. The expression of the enzymes can therefore be manipulated by engineering a small number of regulators that are responsible for the coordinated expression of cellulases. In *T. reesei*, unlike in *Neurospora crassa, Fusarium graminearum* or *Aspergillus* spp., the transcriptional activator XYR1 (Stricker *et al.,* 2006) is the major transcriptional activator of both cellulase and xylanase gene expression. It belongs to the fungal specific class of transcription factors that contain a domain that binds Zn^2+^ ions by six cysteine residues (Zn2Cys6). XYR1 binds to a 5′‐GGCW_4_‐3′ DNA motif (Furukawa *et al*., 2008). Deletion of this gene eliminates cellulase induction by all known inducers, whereas *xyr1* overexpression enhances it (for review see Bischof *et al*., 2016; Gupta *et al*., 2016b; Druzhinina and Kubicek, 2016). Several strategies for manipulation of XYR1 (and of some other transcriptional regulators that will be described below) to enhance cellulase production or render its expression constitutive have therefore been presented (Table 2). Recently, da Silva Delabona *et al*. (2017) showed that a 26‐fold constitutive overexpression of *xyr1* in another *Trichoderma* spp. (*T*. cf. *harzianum*) resulted in the production of an enzymatic complex that exhibited a 25% enhanced hydrolysis rate of sugarcane bagasse during the first 24 h of saccharification. Also, Ellilä *et al*. (2017) constitutively expressed a XYR1 (V821F) mutant (which leads to reduced glucose repression; Derntl *et al*., 2013) in *T. reesei* and obtained increased cellulase and xylanase production on sugarcane bagasse.

**Table 2:** Published examples of improvement of cellulase production in *Trichoderma reesei* by genetic engineering

Gene	Manipulation method	Promoter	References
*xyr1*	Constitutive overexpression	*pki1*	Seiboth, Karimi *et al*. (2012)
	Constitutive overexpression	*tcu1*	Lv *et al*. (2015)
	Constitutive overexpression, and *ace1* downregulation	*pdc1,* downregulation by antisense with the same promoter	Wang *et al*. (2014)
*cbh1*	Promoter engineering	CRE1 binding sites exchanged against ACE2 and HAP2/3/5 binding sites	Zou *et al*. (2012)
*cre1*	Deletion		Nakari‐Setälä *et al*. (2009)
	Truncation		Nakari‐Setälä *et al*. (2009); Mello‐de‐Sousa *et al*. (2014)
*ace1*	Deletion		Aro *et al*. (2003)
*ace3*	Introduction of multiple gene copies		Häkkinen *et al*. (2014)
*lae1*	Constitutive overexpression	*gpd1*	Seiboth‐Karimi *et al*. (2012)
*vel1*	Constitutive overexpression	*tef1*	Karimi Aghcheh *et al*. (2014)

Three other proteins or protein complexes (i.e. the Zn2Cys6 transcriptional activators ACE2 and ACE3, and the CCAAT‐binding protein complex (HAP2/HAP3/HAP5) are also involved in the regulation of cellulase gene expression in *T. reesei*. So far, only the overexpression of *ace3* has been shown to result in enhanced cellulase formation (Häkkinen *et al*., 2014). Other regulators may still be detected: introduction of multiple copies of the gene for the still uncharacterized Zn2Cys6 transcription factor Trire2:80291 into *T. reesei* was also shown to increase CBH1 formation twofold (Häkkinen *et al*., 2014). In addition, cellulase gene expression in *N. crassa* involves several other transcription factors, of which at least CLR2 and VIB1 (Coradetti *et al*., 2012; Xiong *et al*., 2014) have orthologues in *T. reesei* (Trire2:26163 and Trire2:54675, respectively; see genome annotation in Druzhinina *et al*., 2016). However, it is not known yet whether they also regulate cellulase gene expression in *T. reesei*, and – if so – whether their manipulation could be used to improve cellulase production.

Two interesting alternative perspectives for manipulation of cellulase gene expression have recently been presented: Zhang *et al*. (2017) constructed a hybrid cellulase regulator, which consisted of the DNA‐binding domain of the glucose‐repressor CRE1 (see below) fused to the XYR1 effector binding domains. Its overexpression in *T. reesei* led to an approximately 30‐fold increase of constitutive cellulase and hemicellulase production on glucose. Zhang *et al*. (2016b) constructed an artificial zinc finger protein library and expressed it in *T. reesei*. One of the respective transformants showed a 55% rise in cellulase activity against filter paper and an 8.1‐fold increased β‐glucosidase activity.

Cellulase gene expression has also been shown to be repressed by rapidly assimilated carbohydrates, which is due to the action of carbon catabolite repressor protein CRE1 (Nakari‐Setälä *et al*., 2009). CRE1 interferes both with constitutive as well as induced cellulase and hemicellulase gene expression, but the degree of this repression varies for different cellulase and hemicellulase genes (see Druzhinina and Kubicek, 2016 for review). As an example, only the constitutive but not the inductive expression of the cellobiohydrolase gene *cel6A* and of the xylanase gene *xyn1* is repressed by CRE1 (Mach *et al*., 1996; Zeilinger *et al*., 2003). In contrast, CRE1 represses both constitutive and induced expression of the cellobiohydrolase 1 gene *cel7a* (Nakari‐Setälä *et al*., 2009). Elimination of the function of CRE1 had already been obtained by classical mutagenesis and resulted in a strain with increased cellulase formation in the presence of increased concentration of inducing carbon sources (cellulose or lactose; Eveleigh and Montenecourt, 1979). This strain – known as *T. reesei* RUT C30 – bears a truncated version of the *cre1* gene that expresses only the zinc finger but not the transactivating domains (CRE1‐96; Ilmén *et al*., 1996). It is noteworthy that some strains used for the production of cellulases and hemicellulases on the industrial scale are descendants of RUT C30. Recombinant strains of *T. reesei* with *cre1* loss of function have been analysed too, but – in contrast to RUT C30 – they have a pleomorphic phenotype (Nakari‐Setälä *et al*., 2009) and their use in cellulase production is therefore limited.

Another Zn2Cys6 transcription factor – ACE1 – acts as a partial repressor of cellulase and xylanase gene expression, and strains carrying a deleted allele displayed enhanced cellulase formation (Aro *et al*., 2003). The physiological conditions that trigger ACE1 repression are not understood yet. ACE1 is an orthologue of *Aspergillus nidulans* StzA/SltA (Aro *et al*., 2003) that plays a role in Ca^2+^ homoeostasis (Spielvogel *et al*., 2008) and has further been implicated in nitrogen control (Chilton *et al*., 2008). It is however intriguing that the ACE1 orthologue of *Colletotrichum gloeosporioides* is mainly involved in appressorium formation (Dubey *et al*., 2016). An elucidation of the function of ACE1 in *T. reesei* will be essential before *ace1* can be used for science‐based strain engineering.

After this review had been submitted, Cao *et al*. (2017) reported on the identification of a further repressor of cellulase gene expression, RCE1. Disruption of its gene enhanced the induced expression of cellulase genes and led to a significant delay in termination of induction. RCE1 did not participate in CRE1‐mediated catabolite repression, but antagonized the binding of XYR1 to the cellulase promoters.

### Promoter engineering

Manipulation of the binding sites for transcriptional regulators in the cellulase genes is another strategy to modify their expression. Given the number of cellulases produced by *T. reesei*, this can be a time‐consuming process. Nevertheless, replacing the CRE1 binding sites within the *cbh1* promoter by the binding sites for the transcription activators ACE2 and the HAP2/HAP3/HAP5 complex enhanced transcription of a test gene (green fluorescent protein) under cellulase inducing conditions sevenfold (Zou *et al*., 2012). This approach may have its merit when the expression of only a few genes is targeted. On the other hand, engineering the *xyr1* promoter (XYR1 also needs to be induced for cellulase and hemicellulase formation; Lichius *et al*., 2014) – should provide a much more straightforward approach towards enhancement or modulation of cellulase production. Yet no such experiments have been published up to date.

### Epigenetic engineering

Epigenetic engineering has recently been reviewed as a potentially new tool for industrial improvement of fungi (Aghcheh and Kubicek, 2015). The term has been coined by Waddington (1942) for heritable changes in gene expression that do not involve changes in the underlying DNA sequences. In the current understanding, two major levels of epigenetic regulation are important: DNA methylation; and chromatin remodelling by histone modification. While evidence for a regulatory role of DNA‐methylation in *T. reesei* is not available, some aspects of chromatin remodelling have been demonstrated.

Gupta *et al*. (2016b) have recently discussed the remodelling of chromatin by transcriptional activators and emphasized it as an emerging approach for improvement of cellulase formation. Under cellulase inducing conditions, the chromatin packing around the cellulase‐encoding genes *cbh1* and *cbh2* opens, but this does not occur in a *xyr1*‐deletion strain (Mello‐de‐Sousa *et al*., 2015, 2016). Consequently, Mello‐de‐Sousa *et al*. (2015) identified several genes encoding chromatin remodelling enzymes, including histone acetyltransferases, whose expression is significantly different in the ∆*xyr1* and its parental strain. Whether these genes could be used to improve cellulase production has not been tested yet. Another histone acetyltransferase (GCN5), however, has been shown to be necessary for cellulase gene expression, and the acetylations of K9 and K14 of histone H3 in the *cbh1* promoter were strongly reduced in the *T. reesei Δgcn5* strain (Xin *et al*., 2013).

The carbon catabolite repressor CRE1 has also been implicated in chromatin remodelling: strains expressing a non‐functional CRE1 exhibit a loss of positioned nucleosomes within the *cbh1* and *cbh2* genes under repressing conditions only (Zeilinger *et al*., 2003; Ries *et al*., 2014). Interestingly, the truncated version of CRE1 (CRE1‐96) of RUT C30 seems to trigger chromatin opening (Mello‐de‐Sousa *et al*., 2014), likely by regulating the expression of *snf2/htf1* (Portnoy *et al*., 2011), a putative helicase that might participate in an ATP‐dependent chromatin remodelling complex (Mello‐de‐Sousa *et al*., 2014). Identification and engineering of chromatin remodelling proteins are obviously a promising possibility for strain development.

In addition, there is another group of fungal genes that participate in chromatin modification and whose manipulation has been shown to modulate cellulase and hemicellulase production: the Velvet‐LaeA/LAE1 complex (Seiboth, Karimi *et al*., 2012; Karimi Aghcheh *et al*., 2014; Liu *et al*., 2016). LaeA, originally identified in *A. nidulans* as a regulator of secondary metabolite gene expression, has characteristics of an *S*‐adenosyl‐l‐methionine arginine protein methyltransferase (Sarikaya‐Bayram *et al*., 2015). However, LaeA appears not to methylate histones. Rather it seems to function by interaction with the transcription factors of the Velvet protein complex. Constitutive overexpression of both *lae1* as well as *vel1* in *T. reesei* has been shown to enhance cellulase expression and secretion (Seiboth, Karimi *et al*., 2012; Karimi Aghcheh *et al*., 2014).

### Metabolic engineering

A significant body of work has been dedicated to the characterization of the metabolic steps that are involved in the signalling of cellulase gene expression. The current state of knowledge in this area has recently been reviewed (Druzhinina and Kubicek, 2016; Gupta *et al*., 2016b). Despite a fair amount of progress, an essential improvement of cellulase production by the manipulation of individual genes involved in this process has not yet occurred. This is in part due to the fact that manipulation of genes involved in central metabolism and particularly in signal transduction often produce pleiotropic effects that are difficult to use for strain improvement. Whole‐genome dedicated strategies, such as the use of whole‐cell metabolic models, likely offer the best available strategy for the identification of the limiting steps for cellulase production. To this end, Castillo *et al*. (2016) applied the CoReCo (comparative metabolic reconstruction framework) pipeline (Pitkänen *et al*., 2014) for the construction of a high‐quality metabolic model of *T. reesei* (BIOMODELS database). The model contains a biomass equation, reaction boundaries and uptake/export reactions, which make it ready for the simulation of protein production processes. It was applied by Pakula *et al*. (2016) for analysis of the flux balances of its metabolism, using a transcriptomic analysis of protein production by *T. reesei* in chemostat cultivations. The study showed that high protein (cellulase) production might be limited at the level of biosynthesis of sulfur‐containing amino acids, which identifies a clear target for metabolic engineering that was so far not detected by other means.

## The engineering toolbox of *T. reesei*

A suitable molecular tool box that enables easy exchange and manipulation of genes is a prerequisite for engineering *T. reesei* or individual components of its cellulolytic cocktail, as reviewed above. This topic has been subject of several exhaustive recent reviews (Steiger, 2013; Bischof and Seiboth, 2014; Bischof *et al*., 2016; Gupta *et al*., 2016b), and we shall therefore discuss it only very briefly. The low efficiency of gene targeting has for a long time been a major bottleneck in this regard, but was finally – like in several other fungi – solved by inactivating components of the non‐homologous end joining pathway of DNA repair such as *tku70* or *tmus53* (Guangtao *et al*., 2009; Steiger *et al*., 2011; Schuster *et al*., 2012). More recently, Ouedraogo *et al*. (2015, 2016) demonstrated that an I‐SceI mediated double strand break in a *T. reesei tku70* gene improved transformation efficiencies and increased homologous integration up to 90–100%. Also the CRISPR (clustered regularly interspaced short palindromic repeats)/Cas9 system (D'Agostino and D'Aniello, 2017) has been adapted for use with *T. reesei* (Liu *et al*., 2015). It depends only on the Cas9 (CRISPR‐associated) nuclease which uses a single chimeric guide RNA for targeting, and so introduces specific DNA double strand breaks that stimulate gene targeting.

Further, a number of new approaches were offered that allowed the insertion of expression cassettes at defined genomic regions. Derntl *et al*. (2015) constructed *T. reesei* strains bearing truncated versions of several genes resulting in auxotrophic phenotypes, and tested the rescue of prototrophy by transformation with a complementary fragment of the same gene. Among them, they used the *ade2* locus (encoding a phosphoribosylaminoimidazole carboxylase necessary for purine biosynthesis) because integration into this locus destroys *ade2,* the *Δade2* disruptants accumulate polymerized 5‐aminoimidazole ribonucleotide (Ugolini and Bruschi, 1996) and can therefore easily be identified by the red colour. Derntl *et al*. (2015) also successfully demonstrated the applicability of the *asl1* (encoding argininosuccinate lyase of arginine biosynthesis), the *hah1* (encoding homoaconitate dehydratase involved in lysine biosynthesis) and the *pyr4* genes (encoding the orotidine‐5‐phosphate decarboxylase of the uridine biosynthesis pathway) for this approach to obtain site‐directed integration in *T. reesei*.

Even if the transformation frequency is high, identification of the transformants that express the target protein at desired levels can still be a tedious task because the expression levels between transformants can be very variable. Subramanian *et al*. (2017) have therefore adapted the 2A peptide system from the foot‐and‐mouth disease virus to *T. reesei*. This tool allows multiple independent genes to be transcribed as a single mRNA. Upon translation, the 2A peptide sequence causes a ‘ribosomal skip’ between its two C‐terminal amino acids and generates two independent gene products. A target protein can therefore be cotranslated with a readily testable marker protein, and colonies with desired expression levels can so be identified. The authors tested this by coexpression of CEL7A and an enhanced green fluorescent protein as a marker and obtained similar levels of expression of both proteins. This system therefore offers an efficient strategy to test the expression of heterologous proteins in *T. reesei*, and provides a novel platform for multi‐protein‐expression in approximately equimolar ratios using a single polycistronic gene expression cassette.

A further essential tool needed for strain engineering is promoters that allow high constitutive expression without dependence on special physiological conditions. An important issue is thereby that the transcriptions factors that bind to the respective regulatory elements in these promoters are present in sufficient quantities in the cells because their use would otherwise be limiting the expression by titration effects. Table 3 summarizes such promoters and their use in *T. reesei*.

**Table 3:** *Trichoderma reesei* promoters used

	Promoter	Condition	
Constitutive expression	Glycolytic genes *(pki1, gpd1, pgd1, eno1, pdc1*		Li *et al*. (2012); Seiboth, Karimi *et al*., (2012); Wang *et al*. (2014); Linger *et al*. (2015)
	*cre1:xyr1* hybrid		Zhang *et al*. (2017)
	*cDNA1*		Uzbas *et al*. (2012)
	*tef1*		Seiboth, Karimi *et al*. (2012)
Inducible expression^a^	*tcu1* copper transporter	Copper deficiency	Lv *et al*. (2015)
	TauD‐dioxigenase	Methionine deficiency	Bischof *et al*. (2015)

a Promoters of cellulase and xylanase genes are not listed.

Also, there is a demand for promoters that could be temporarily turned either on or off. Traditionally, the *T. reesei cbh1* and *xyn1* promoters (of the genes encoding the cellobiohydrolase CEL7A and the endo‐β‐1,4‐xylanase XYN1, respectively) have been used for this purpose, but the respective inducers or repressors also act on other cellulases and xylanases and are therefore not sufficiently specific (for review see Bischof and Seiboth, 2014). To solve this problem, Bischof *et al*. (2015) introduced the promoter of a gene encoding Trire2:123979 (a putative dioxygenase of unknown function) which in *T. reesei* can be strongly repressed by addition of 0.1 mM l‐methionine. An alternative strategy was presented by Lv *et al*. (2015), using the promoter of the gene encoding the copper transporter TCU1 (Trire2:52315), which can be repressed by Cu^2+^ ions of > 0.2 μM. This system was recently used to manipulate the expression of the histone acetyltransferase GCN5 (Trire2:64680) in *T. reesei* (Zheng *et al*., 2016).

Towards the development of an inducible expression system, Zhang *et al*. (2016a) constructed a modular synthetic regulator by fusing the gene fragments encoding the DNA‐binding domains of yeast Gal4, the light responsive domain of *N. crassa* Vivid, and the *Herpex simplex* VP16 transactivation domains. The fusion gene was put under the control of a synthetic promoter carrying five copies of the Gal4 consensus binding site. It was successfully expressed in *T. reesei* in the presence of light. However, the operation of this system at an industrial scale with high‐density suspensions of cellulosic substrates and fungal mycelium has not been tested yet.

## Conclusions

The potential of lignocellulosic biomass as renewable feedstock for substituting a significant fraction of today's fossil fuel consumption has been an attractive theory for many years. In recent years, the first industrial‐scale cellulosic ethanol plants have started to operate. Yet competition with technologies based on fossil resources is still hampered by the high costs for the pretreatment and enzymatic saccharification steps due to the natural recalcitrance of lignocellulosic biomass. In this article, we have focused on saccharification, and discussed how the production and properties of the cellulases from the major industrial producer *T. reesei* could be changed towards a better process economy. In this regard, it is interesting to note that – despite the amount of work dedicated to the understanding of the catalytic mechanisms of some of the major cellulases – comparatively little work has been published that shows whether the introduction of activity increasing mutations indeed lead to strains that produce better enzyme preparations. Thereby it should also be borne in mind that cellulases function at a solid–liquid interface of a physically and chemically heterogeneous substrate, i.e. native or pretreated plant cell walls, which clearly impact the activity of the enzyme, and makes the enzyme dependent on other enzyme partners. Finally, it is still an open question whether (and if so why) the multiplicity of some cellulases (e.g. the endo‐β‐1,4‐glucanases) and the LPMOs are essential for the overall hydrolysis of commercially used lignocellulose substrates. Answers to these questions are needed for genetic engineering of *T. reesei* strains to produce more active cellulase mixtures.

Engineering of cellulase production is a related, yet different issue: industrial strains producing extremely high cellulase concentrations (over 120 g l^−1^ total protein) have been reported (Gupta *et al*., 2016a), and it is worth of discussion whether a further increase (or shortening of the fermentation time needed to achieve this) is feasible and required. Smaller companies or newcomers may, however, want to construct their own producer strain, and can now select from a variety of conditions for this purpose. Also, the carbon source for production of the enzymes needs not to be the same that is used for saccharification, and the producer strain, therefore, may have to be tailored to form cellulases on media not containing lignocellulose or containing a mixture of lignocellulose and other carbon sources. Unfortunately, most of the alterations achieved by metabolic engineering, promoter or transcription factor engineering and epigenetic engineering have been tested only under laboratory conditions, frequently using minimal media, and by only measuring the relative abundance of cellulase mRNA. The presence of a heterogenous polymer and the chemical changes that occur in the medium during its hydrolysis will likely have a strong impact on the function of these engineered strains. It is therefore difficult to predict whether the introduced mutation would indeed by beneficial for the industrial process. We strongly recommend that such engineered strains should always also be tested on proper lignocellulose substrates. In addition, an *in vitro* saccharification experiment with the enzymes produced in these cultivations should also be included.

## Conflict of interest

None declared.

## Supporting information


**Table S1.** 100 best hits in a blastp analysis* using the *H. insolens* cellobiose dehydrogenase as a query.
